# Beyond gender mainstreaming: transforming humanitarian action, organizations and culture

**DOI:** 10.1186/s41018-023-00138-1

**Published:** 2023-04-22

**Authors:** Geeta Rao Gupta, Caren Grown, Sara Fewer, Reena Gupta, Sia Nowrojee

**Affiliations:** 1 Girls and Women Strategy, United Nations Foundation, Washington, DC USA; 2 Center for Sustainable Development, The Brookings Institution, Washington, DC USA; 3 The Royal Swedish Academy of Sciences - Kungliga Vetenskapsakademien, Stockholm, Sweden; 4 Girls and Women Strategy, United Nations Foundation, Washington, DC USA

**Keywords:** Humanitarian programming, Humanitarian culture, Gender inequalities, Gender mainstreaming, Results-based approaches

## Abstract

The United Nations and major humanitarian organizations have made policy commitments to promote gender equality and empower women and girls. This study assesses the extent to which humanitarian responses have met these commitments based on reviews of gender mainstreaming, textual analysis of policy and program cycle documents, and interviews with humanitarian actors. The analysis reveals that while gender mainstreaming may raise awareness and make fixes at the margins, its focus has been limited to altering internal processes rather than emphasizing results for women and men and girls and boys. Our study also analyzes the cultural and institutional context in which gender mainstreaming takes place. The culture of humanitarian organizations has been characterized as hierarchical and driven by a short-term crisis response with a distinctly macho style of functioning, which is misaligned with gender mainstreaming. We propose replacing gender mainstreaming with a results-focused approach rooted in behavioral science that uses evidence of the conscious and non-conscious drivers of human behavior to address problems, alongside other efforts to change the internal culture of humanitarian organizations.

## Introduction

An increasing number of people today live in countries that are marked by internal civil conflict and war; by 2030, more than half of the world’s extreme poor are projected to live in countries marked by fragility, conflict, and violence (World Bank Group 2020).

Ample evidence shows that conflict affects girls and women differently than boys and men, especially related to capabilities, which refers to basic human abilities developed through health, nutrition, and education; opportunities, which refer to access to economic resources and assets; safety, which is the assurance of security and freedom from violence; and agency, which is an individual’s ability to make decisions about strategic life outcomes and to have voice in governance and political processes (Kabeer 1999; Grown et al. 2005; Hudson et al. 2009; Buvinic et al. 2013; Mirzazada et al. 2020; Save the Children 2022).

Recognizing the gender differentiated impacts of conflict, the United Nations Inter-Agency Standing Committee (IASC) for Humanitarian Coordination, calls for: “…the need to understand the specific needs, capacities and priorities of women, girls, boys and men, and integrate this understanding throughout the programme cycle…facilitate the active participation and leadership of women and girls in humanitarian action and beyond; and promote transformative change for more inclusive and equitable societies” (IASC Reference Group on Gender and Humanitarian Action 2018)*.*

Despite the best of intentions, several recent reviews reveal that the ambition expressed in that statement has not been achieved. This is primarily because gender mainstreaming, which is the approach the humanitarian sector has taken to address gender inequalities, has not been effective (Pillay 2018; Lokot 2021; Daigle 2022). Our study draws on reviews of gender mainstreaming, textual analysis of policy and program cycle documents, and interviews with humanitarian actors and experts to explore why this is so and offers reflections on a way forward. The analysis reveals that while mainstreaming may raise awareness and fix things at the margins, its focus has been limited to altering internal processes rather than emphasizing results for women and men and girls and boys.

Our study also analyzes the cultural and institutional context in which mainstreaming takes place. Importantly, the culture of humanitarian organizations has been characterized as hierarchical and driven by a short-term crisis response with a distinctly macho style of functioning, which is misaligned to a gender mainstreaming approach (Thoretz 2019; Lokot 2021). Such a culture reinforces cognitive biases and behaviors that are not easily amenable to change. Research in social psychology and evolutionary anthropology has demonstrated that these behavioral biases are driven by a deep, social logic allowing humans to efficiently learn from, adapt to, and reinforce the norms of their social environment (Tomasello et al. 2005; Frith and Frith, 2011). Taken together, literature from behavioral and organizational science suggests that “decision making within organizations will be heavily influenced by the established (often unconscious) patterns of behavior and social norms of that organization’s culture” (Hallsworth and Kirkman 2020).

Using qualitative analysis, this paper concludes that ultimately, what is needed to effectively institutionalize and sustain an alternative approach—one that uses evidence of the conscious and non-conscious drivers of human behavior to address problems, alongside efforts to change the internal culture of humanitarian organizations—will be more effective in achieving gender equality and empowering women than gender mainstreaming.

The next sections of the paper are structured as follows. We begin with a review of the literature on gender mainstreaming in development and in humanitarian action, followed by a discussion of our methodology to analyze the current response to gender inequalities in conflict settings. We then present our findings, and finally, we conclude with suggestions for a different approach for the sector.

## Review of gender mainstreaming in development and humanitarian response

Over the past three decades, the IASC has issued policy statements and guidance to integrate a gender perspective in humanitarian response. The policy position has evolved from a somewhat narrow focus on the specific needs and vulnerabilities of women and girls and the imperative to uphold their human rights (IASC 1999), to a broader focus on the empowerment of women and girls (IASC 2008), and most recently, a more expansive call to transform gender roles and norms (IASC 2017).

Throughout this evolution, gender mainstreaming has remained the dominant approach. Gender mainstreaming involves assessing and differentiating the implications for women and men of any planned action, including legislation, policies, and programs, in all areas and at all levels (ECOSOC 1997). Typically, mainstreaming consists of an organizational structure based on a hub-and-spoke model, with a central gender unit responsible for developing a gender policy and supported by gender focal points placed in departments/offices across the organization that provide technical support to implement the policy. This often includes training, as well as drafting tools such as checklists and guidance on how to mainstream.

As discussed in the development literature, gender mainstreaming has failed to produce results in reducing gender disparities or in transforming an organization’s approach to programming for women’s empowerment but instead has become a “tick-the-box” (or add-on) exercise without achieving meaningful outcomes (Mehra and Rao Gupta 2008; Ravindran and Kelkar-Khambete 2008; Madsen 2011; Meier and Celis 2011). Guidance is often generic, with too many handbooks and checklists, and gender specialists are more frequently located in headquarter than country offices. Efforts to build capacity among staff have, by and large, focused on increasing general awareness about “gender” rather than on the technical skills and knowledge needed to impact outcomes in and across sectors. 

Moreover, because the mainstreaming approach has generally not been supported by evidence and concrete solutions, the work of gender experts is seen as empty advocacy. To make matters worse, gender experts are viewed as the “gender police.”

Given the lack of technical sector expertise, monitoring and evaluation of gender mainstreaming mostly focuses on the implementation of the approach, rather than on the results achieved (Rao and Kelleher 2005; Moser and Moser 2005; Madsen 2011) and insufficient reporting of results from the field makes it difficult to learn from challenges or successes.

Beyond these issues, the term “gender” itself has triggered resistance and fatigue in development organizations. As noted in the findings, “gender” is often seen as a euphemism for women, which is understandable since investments for gender equality often target girls and women to rectify the historical legacy of disadvantage they face. However, reducing the word “gender” to mean women misses the relational context and unequal power structures between men and women and the ways in which gender norms are embedded in institutions and social/cultural interactions.

Many of the limitations of gender mainstreaming and how it has been implemented in development organizations, as highlighted above, also characterize the humanitarian sector (Pillay 2018; Thoretz 2019; Lokot 2021). Indeed, the recent IAHE GEEWG report also notes that the experience of gender mainstreaming in humanitarian organizations has faced similar challenges as the development field (IASC 2020a). Analysts also point out some humanitarian sector-specific limitations. Gender equality is not always seen as the first priority in responding to a complex emergency but rather is seen as a longer-term development mandate (Thoretz 2019; Hart, 2021). Humanitarians see their focus first and foremost on life-saving interventions and meeting immediate needs, arguing that in volatile contexts it is difficult to move from meeting basic needs to addressing larger-scale social change (Hart, 2021; Daigle 2022). Others have pointed out that the short-term nature of the project cycle also mitigates against longer-term change efforts (Hart, 2021). Finally, for some in the field, gender equality is seen as antithetical to the humanitarian principles of neutrality and impartiality because changing the power imbalances between women and men is seen as interfering with cultural norms (Santos 2020; Daigle 2022).

As noted above, the word “mainstreaming” is defined to mean that attention to gender will be integrated in every sector and every intervention. Below we will argue that a results-focused approach helps to prioritize actions and is more effective than doing everything everywhere. Moreover, the reviews of gender mainstreaming attribute the lack of effectiveness to limited expertise, funding, accountability and compliance mechanisms, and commitment by stakeholders (Rao and Kelleher 2005; Thoretz 2019; Hart, 2021; Daigle 2022). Therefore, they recommend strengthening and increasing access to gender equality expertise, improving planning and monitoring processes, enhancing management and accountability, and improving the tracking of resources. As we also discuss below, those are not the only reasons for the failure of gender mainstreaming. We argue that not only is mainstreaming itself a flawed approach, it is also unrealistic to think it can succeed in a humanitarian culture that is built upon deeply patriarchal norms.

## Methodology

In light of these issues, the goal of our qualitative analysis was to determine the extent to which the ambitions of the IASC and other humanitarian actors with respect to mainstreaming are being met. Specifically, we assessed how the humanitarian response addresses differences and inequalities in capabilities, opportunities, safety, and agency between women and men, girls and boys in eight conflict-affected countries, through a review of key policy and program documents and key informant interviews with representatives of select organizations.

Our study focused on humanitarian activities; both short-term emergency responses to acute and protracted armed conflicts and managing and meeting the needs of displaced populations in refugee camps or host communities as part of medium- and long-term recovery and resilience building.

We selected fifteen organizations including multilateral institutions, bilateral donor agencies, development banks, and international non-governmental organizations, with varying histories of instituting gender policies and operating in different geographies (Table 1). These 15 organizations were selected based on a desk review and availability of current and/or former staff to participate in key informant interviews. Five organizations are dedicated solely to humanitarian work, while the remaining 10 have a dual mandate to work on both development and humanitarian assistance.

**Table 1:** Sample of humanitarian organizations

**Organization type (key informant ID)**^**a**^	**Organization**
United Nations (UN1, 2…14)	• Office of the Coordinator for Humanitarian Affairs (OCHA)^b^ • United Nations High Commissioner for Refugees (UNHCR)^b^ • United Nations Children’s Fund (UNICEF) • World Food Program (WFP) • World Health Organization (WHO)
Bilateral donor agencies (BDA1, 2…9)	• Global Affairs Canada • Swedish International Development Cooperation Agency (Sida) • US Agency for International Development (USAID)
International Finance Institutions (IFI1, 2…4)	• African Development Bank (AfDB) • European Bank for Reconstruction and Development (EBRD) • Inter-American Development Bank (IDB)
International non-governmental organizations (NGO1, 2…9)	• International Federation of Red Cross and Red Crescent Societies (IFRC)^b^ • International Committee of the Red Cross (ICRC)^b^ • International Rescue Committee (IRC)^b^ • CARE
Other Independent Experts (OE1, 2…8)	

^a^To ensure confidentiality, key informants were assigned a serial number within each category of type of organization. In the text below, we use the assigned serial number to cite the key informants

^b^Organizations that only provide humanitarian assistance

We first analyzed the institutional documents that offer guidance on how to advance gender equality and the empowerment of women in humanitarian settings, including the IASC Gender Handbook, sectoral guidance and minimum standards issued by global sectoral clusters, and organizational policies, strategies, and guidance from the global humanitarian organizations that were the focus of this study. We sought to understand the expectations and standards for “gender mainstreaming” in humanitarian programming overall and within each organization.

We also reviewed humanitarian program cycle documents for the most recent year available for eight countries representing different regions and stages of conflict: Afghanistan and Bangladesh in Asia; Yemen and Syria in the Middle East; South Sudan and Nigeria in sub-Saharan Africa; and Colombia and Venezuela in Latin America. The documents reviewed for each country were the Humanitarian Needs Overviews (HNO), Humanitarian Response Plans (HRP), Humanitarian Annual Reports (HAR), and humanitarian appeals. We identified whether the materials discussed needs and gaps in safety and security, basic needs and capabilities, opportunities, and agency for women as compared to men; or facilitated the active participation and decision-making of women and girls, in Health, Education, WASH, Shelter, Logistics & Camp coordination and management, Protection and Gender Based Violence, Early Recovery and Livelihoods, and Food Security and Agriculture. We then tracked whether the needs identified in the HNOs were addressed in the HRPs and reported on in the HARs. To the extent available, we also reviewed Financial Tracking Service data for the years in which the country documents were written to identify the amount of humanitarian funding requested and received for gender-based violence and gender mainstreaming.

Additionally, we conducted interviews with a purposive and snowball sample of 44 individuals from 15 global organizations (Table 1)^1^, who are responsible for shaping their organization’s response to gender differences and inequalities in humanitarian settings. Our initial set of key informants was also asked to suggest additional contacts to participate in the study. The sample size was determined by data saturation: interviewing enough key stakeholders from an organization to reach an understanding of each organization’s approach to gender mainstreaming.

Finally, we interviewed independent experts with experience in gender mainstreaming and organizational change.^2^ The questions we asked covered six themes related to each organization’s response to gender inequalities and the empowerment of women in humanitarian operations*.* Interviews were coded according to a preliminary list of codes derived from the analytic framework (Appendix 1). These codes were adapted and expanded based on topics and patterns emerging from the interviews. The interviews were coded independently by two of the authors, and results were jointly discussed to develop a summary of the codes and related themes.

## Findings

Overall, we find that despite high aspirations and many valiant efforts, the humanitarian sector is falling short of its commitment to promote gender equality and empower women and girls. Our key informant interviews and review of organizational policies, technical resources, and humanitarian program cycle documents revealed five key findings on gender mainstreaming and three key findings pertaining to the culture of and behavior in humanitarian organizations and programs (Table 2).

**Table 2:** Summary of key findings

**A. Gender mainstreaming: a flawed approach**
(1) A gap between intention and implementation
a. Inconsistent quality of SADD and reporting on gender differences and inequalities
b. Lack of linkage between documents across the humanitarian program cycle
(2) A focus on process, not results
(3) Conceptual confusion and inadequate technical resources
(4) Basic needs, protection, and participation prioritized over GBV, economic opportunities, and agency
a. Attention to basic needs and capabilities
b. Neglect of girls’ and women’s economic opportunities
c. Women’s and girls’ inclusion limited to representation rather than decision-making
(5) Inadequate technical and financial resources
a. Inconsistent technical resources across organizations and sectors
b. Inadequate gender expertise
c. Insufficient financial resources
**B. Humanitarian culture and behavior: a barrier to change**
(1) Savior mentality and a macho culture
(2) A humanitarian culture that tolerates abuse
(3) Short-termism

### Gender mainstreaming: a flawed approach

Above, we noted briefly, the limitations of gender mainstreaming that have been found in the development and humanitarian literature. Our review reinforced these findings and distinguished five specific issues: the gap between intention and implementation; a focus on process, not results; conceptual confusion; basic needs priorities over economic opportunities; and inadequate technical and financial resources.

#### A gap between intention and implementation

As part of gender mainstreaming, the IASC recommends that a gender analysis be conducted at all stages of an emergency response (IASC Reference Group on Gender and Humanitarian Action 2018) to help humanitarian actors better understand the affected population, examine the impact of the emergency, and verify that the humanitarian response meets the distinct needs and priorities of women, girls, men, and boys. Organizations throughout the humanitarian sector are aligned with this recommendation: 12 of the 14 organizations we studied require conducting gender analyses (Appendix 2). However, we found significant variation in the quality of data as well as gaps in addressing gender inequalities across phases of the program cycle in our review of information in the HNOs, HRPs and HARs.

##### Inconsistent quality of SADD and reporting on gender differences and inequalities

Sex and age disaggregated data (SADD) are used frequently, but not comprehensively, across the humanitarian program cycle documents, to describe the number of people in need or reached by a particular sector^3^. For example, of the HNOs, HRPs, and HARs examined in eight countries, SADD was used in 19 of 24 Education Cluster descriptions and 17 of 24 Shelter Cluster descriptions. However, there were a few notable gaps. For example, the Yemen 2019 HNO included SADD by sector, as well as by various categories of need, but in the HRP and HAR, no SADD were reported (UN OCHA and UN Country Team in Yemen 2019; UN OCHA 2020a, b). For Colombia, SADD was missing in the 2019 HNO, included in the HRP, and then used for only some sectors in the HAR (UN OCHA 2018). Key informants explained that staff may lack resources or training to gather SADD in conflict-affected settings and that SADD is sometimes overlooked unless the Humanitarian Country Teams insist on it (OE5, OE6, UN3, UN7, UN11, UN14).

We found greater inconsistencies in the extent to which humanitarian program cycle documents identified specific gender differences and inequalities in access to services (Table 3). For example, six of eight HNOs in the Education Cluster identified that girls have less access to education than boys; however, only five of the eight HRPs and four of the eight HARS included any mention of such differences. In the Shelter Cluster, five of the eight HNOs identified gender differences in shelter-related needs, such as barriers facing women in accessing shelter assistance, but only four of the eight HRPs and two of the eight HARs discussed such differences. The same trend is apparent in other sectors.

**Table 3:** Identification of gender differences and inequalities in humanitarian program cycle documents, by country and sector

	**Education cluster**			**Shelter cluster**		
	**HNO**	**HRP**	**HAR**	**HNO**	**HRP**	**HAR**
**Afghanistan**	✓	✓		✓		
**Bangladesh (Rohingya Refugee Response)**	✓	✓			✓	✓
**Colombia**	✓	✓		✓	✓	
**Nigeria**	✓		✓	✓		
**South Sudan**		✓	✓		✓	✓
**Syria**	✓		✓	✓	✓	
**Venezuela**	✓	✓				
**Yemen**	✓			✓		

Note: ✓ = document identified gender differences and inequalities; *HNO* Humanitarian Needs Overview; *HRP* Humanitarian Response Plan; *HAR* Humanitarian Annual Report; *SADD* inclusion of sex and age disaggregated data

The quality of the information provided also varied greatly. In the Education Cluster, for example, gender differences focused on reasons why girls are held back from school, such as family concerns about girls’ safety walking to school, a lack of sex-segregated toilets at schools, and child marriage. Only two of the eight HNOs identified factors impacting boys’ education, such as needing to work and contribute to family income.

##### Lack of linkage between documents across the humanitarian program cycle

In the flow of the humanitarian program cycle, the HNOs should be used to inform the HRPs, and the HARs should report on progress in implementing the HRPs. However, our review revealed that HRPs and HARs did not regularly reflect the needs identified in the HNOs. For example, for the Education Cluster, documents for only three of the eight countries (Bangladesh, Nigeria, and Syria) identified the need for sex-segregated WASH facilities and responded to that need in the respective HRPs and/or HARs. Although similar needs were identified in the remaining HNOs, they were not carried through in the HRPs, nor reported in the HARs. Similarly, the Syria HNO explained that women face barriers to shelter assistance because they lack the necessary civil documentation, yet the corresponding HRP and HAR did not describe if or how this was being addressed.

Overall, it appeared that the analyses and actions at different stages of the humanitarian program cycle were not linked. Most of the documents that described gender differences or inequalities did so at independent points in time, providing narrow snapshots without an explanation of whether or how those inequalities were addressed during implementation.

Key informants elaborated that while gender analyses have increased in recent years, this step is often overlooked, conducted poorly, or not used to inform humanitarian response (BDA7, NGO2, NGO4, NGO9, OE7, UN13, UN14). In the words of one, “Headquarters says we need to have a protection strategy including gender elements, so we’ll call a consultant. A consultant will write a strategy without really understanding, and we’ll have something that sits on a shelf, and that’s where it will stay” (OE7). Another interviewee said, “I’ve seen in refugee camps, they have specific programs for vulnerable women, but there’s no analysis. ... You might have a gender analysis in the overall [fundraising] appeal, but then in the refugee camp they rush to find a couple of women and do a project. ...the ones who are responsible for the area don’t do a general gender analysis” (BDA7).

#### A focus on process, not results

Overall, our analysis revealed that the humanitarian sector assesses success by monitoring processes rather than results for women and men. For example, in 2018, the UN System-Wide Action Plan on Gender Equality and the Empowerment of Women (UN-SWAP 2.0) focused on monitoring gender mainstreaming by using a set of process indicators for results-based management, oversight, accountability, human and financial resources, capacity, and knowledge communication (ECOSOC 2020; UN Women 2020). The lack of focus on results was characteristic of the program response overall.

The emphasis in the annual reports was on the number of sex and age disaggregated beneficiaries of services provided and activities implemented, rather than on closure of gaps between women and men or the difference the intervention made for women and girls compared to some baseline. This made it difficult to capture results. For example, in Afghanistan, the HAR results included the number of women provided with infant feeding services, number of women reached through feedback mechanisms on the humanitarian response, and number of women and girls served through women-friendly health spaces (UN OCHA 2019b). As one interviewee reported, “Currently, we have project completion reports, we count our outputs and outcomes and so on, but not impact. The impact we get is out of countries when they report in their national development plan and so on. For us as an institution it is something that we really need to put some resources [behind]” (IFI2).

Interestingly, several evaluations highlight the need for increased evidence and better monitoring (WHO 2011; WFP 2014; Evaluation Office 2019; UNHCR 2020). In the words of a key informant, “While we’ve had more traction around processes like gender analysis, around thinking about gender issues at the design or startup phase of the project, we really have very little in terms of program evaluations, overall sector evaluations, that I can share with you to say we’ve progressed in this area, and we haven’t progressed in that area” (NGO9).

Focusing on processes rather than results makes it difficult to motivate humanitarian professionals, who are generally driven to achieve results, to become champions of this agenda (IFI4, NGO9, UN3, UN6, UN14). One informant noted, “That’s where we’ve been challenged as a sector because we’ve been met with skepticism. While it’s good to lean on the do-good and do-better mentality of our sector, I actually think what the sector needs is very clear evidence that you actually get better outcomes overall [when you integrate gender]” (NGO9).

#### Conceptual confusion

A third key finding is the lack of conceptual clarity in many of the terms used in institutional documents, and vagueness in what actions to take, and an unmet demand for specific expertise to tailor implementation in different contexts.

Throughout the humanitarian and development sectors, a multitude of “gender” terms are used, such as gender-responsive, gender-sensitive, gender-balanced, gender intentional, and gender-transformative. Often these terms are not clearly defined or even used consistently across organizations, policies, or guidance. As several informants highlighted, the term “gender” itself is interpreted to be code for women and girls as opposed to the structural inequalities that historically have disproportionately disadvantaged women and girls (GDA7, NGO5, NGO9, UN6, UN13, UN14). “[Addressing gender] often means you have to be focused on doing things differently for women and girls. But it’s not about gender equality being only good for women and girls. That’s still an issue that we continue to help staff to think about and understand” (NGO9).

Text in program cycle documents frequently parroted the language in gender policy guidance rather than adapting the guidance to specific contexts. For example, HNO descriptions of gender inequalities were frequently vague, using phrases such as “lack of gender inclusiveness” and “socio-cultural barriers that limit women’s autonomy”. In four of the eight HRPs reviewed, these vague “gender” phrases were used without details on what they meant or how organizations should respond.

This mirrors findings by the IASC on the use of the Gender with Age Marker (GAM), a tool to examine and monitor the extent to which programs address gender and age-related differences (IASC GenCap 2018). Over 11,000 projects have applied the GAM from 2018 to 2020 (IASC 2020b), a step in the right direction. At the same time, several of these projects misunderstood the terms used in the GAM, including “gender mainstreaming.” One key informant explained: “...the issue is that we don’t have a common definition [for gender mainstreaming]..I think the people don’t always know what do we mean by gender mainstreaming, what exactly are we meant to do…. There are different definitions, meanings…. It’s taken for granted that we all know what it means” (GDA1).

#### Basic needs, protection, and participation prioritized over GBV, economic opportunities, and agency

As noted earlier, women and men differ in capabilities, access to economic opportunities, safety, and agency. The frameworks and policies in the humanitarian sector cover all these four areas. The IASC Policy on Gender Equality and the Empowerment of Women and Girls (GEEWG) in Humanitarian Action, for instance, requires all humanitarian actors to meet the specific needs (health and nutrition, education, shelter, food and livelihoods) and priorities of women, girls, men, and boys; prevent, mitigate, and respond to GBV and sexual exploitation and abuse (SEA); and “tangibly promote the meaningful and safe participation, transformative leadership and collective action of women and girls of all backgrounds” (IASC Reference Group on Gender and Humanitarian Action 2017).

Meeting basic needs, such as the provision of water, shelter, and food, and the building of capabilities, such as health and education, is common across program cycle documents. While protection is systematically included, GBV was referenced less often despite the fact that GBV can increase during and just after a crisis. Attention to providing economic options for women and building their agency through opportunities for decision-making were largely missing.

##### Attention to basic needs and capabilities

The humanitarian program cycle documents in all eight countries consistently described a common set of basic needs for girls and women, with slight variation for context-specific factors. For example, in the health sector, each of the eight countries had at least one program document that described specific health needs for women relating to maternal and reproductive health and barriers to antenatal, obstetric, and family planning services. For the WASH sector, all eight countries also had a program document that described safe WASH facilities for girls and women (e.g., improved lighting, locks on latrines) and access to menstrual hygiene management products.

A few examples highlighted the needs of differentiated groups of women, such as displaced ethnic minority groups (Colombia), female heads of households (Afghanistan, Bangladesh, Nigeria, Syria, and Yemen), and pregnant and lactating women (in the nutrition sector in all eight countries), but for the most part women and girls were referenced as a homogenous group. Gender differences in basic needs for boys and men were largely unmentioned.

##### Neglect of girls’ and women’s economic opportunities

Economic opportunities for women and girls did not have the same emphasis as protection in program cycle documents, perhaps because developing economic opportunities is not seen by most humanitarian actors to be their remit, especially if refugee settlements are thought to be a short-term, temporary measure. As one key informant explained: “When I arrived, …there was a lot of building of schools, houses, markets, [and] toilets. We started looking at what are the causes: Why are people living in poverty? People said, “this is a humanitarian situation; you can’t do that kind of programming.” I said, “Well this has been going on for how many years, and yes, we are still doing the life-saving food work. But what about this other stuff?” (NGO4). In 2019, 15.7 million refugees (77% of the refugees under UNHCR’s mandate) had been displaced for at least five consecutive years and were living in protracted refugee situations, making investments in economic activity essential (UNHCR 2019).

In the rare cases when economic issues were raised, the documents lacked explanations of the constraints and suggestions for how they could be addressed. For example, the Colombia 2017 HRP said that women and girls were included in income generation strategies but did not offer details on the strategies (UN OCHA 2016). The Yemen 2019 HAR asserted that women’s and girls’ economic empowerment is critical but did not explain how this was being supported (UN OCHA 2020b).

In the instances where livelihoods were mentioned, the strategies reinforced gender-stereotypical roles for girls and women. For example, in Bangladesh, skill development sessions for women and youth refugees focused on block printing, catering, handicrafts, and tailoring. Key informants expressed frustration that humanitarian strategies miss opportunities to expand women’s economic roles: “Nobody is at all aware of women’s economic roles and their economic needs. I do remember I got to see these economic projects, and they’re teaching women to make soap for an income generating activity. That’s the kind of response that you want to try to change and influence” (OE1).

##### Women’s and girls’ inclusion limited to representation rather than decision-making

Repeated references were made in the program documents to increasing women’s representation and participation in a range of processes. However, only a few examples were found of actions to support women’s agency and decision-making through community engagement. The best example comes from Bangladesh where the HNO noted that women were not represented in decision-making structures, such as community committees for site management and development (International Organization for Migration et al. 2020a). The subsequent HRP described plans to increase girls and women’s representation, including by training women in camp management (International Organization for Migration et al. 2020a). The HNO also identified that women are underserved by engagement and communications strategies. Plans to rectify this in the HRP included a communications strategy to engage girls and women through community centers, increase their participation in community feedback committees, and improve their access to complaint and feedback mechanisms.

#### Inadequate and inconsistent technical and financial resources

Ironically, despite the rhetoric at the highest levels of the humanitarian community on the centrality of gender equality for an effective humanitarian response, funding for the issue lags far behind. Additionally, despite the abundance of technical guidance and training, humanitarian actors continue to request clear guidance on how to address gender inequalities.

##### Inconsistent technical resources across organizations and sectors

As noted earlier, organizations across the humanitarian sector have developed multiple resources and technical guidance to support staff, especially those without knowledge of relevant gender gaps (Appendix 2). However, key informants reported that despite the available tools, staff struggle to translate the information into practice (UN11, UN13): “They are sensitized through training and some documents. But when it comes to operationalizing [guidance to address gender differences and inequalities], to put it into practice, it makes it difficult. In theory, it’s in documents, people are trained, but to operationalize [the guidance] there are further steps to be done, and we have to make it obvious in our different planning documents” (UN11).

In addition to the IASC Gender Handbook, other guidance used by humanitarian workers include the Sphere Handbook and minimum commitments or standards set by the humanitarian clusters.^4^ The Sphere Handbook includes minimum quality standards for different sectors (WASH; food security and nutrition; shelter and settlement; and health) that were developed through broad consensus (Sphere Project 2018). Although not legally binding, the Sphere standards are widely used throughout the sector to guide humanitarian response (Sphere Project 2018).

While the standards include reference to gender equality in humanitarian response, the guidance ranges greatly in quality. What makes using the guidance particularly challenging is that the content ranges from being too *specific* to being too *generic* (Table 4).

**Table 4:** Analysis of quality of gender equality guidance in humanitarian resources, standards, and minimum commitments for key clusters

**Cluster/sector**	**IASC Gender Handbook**	**Sphere standards**	**Cluster minimum commitments/standards**
**Health**	Specific	Generic	Generic
**WASH**	Specific	Specific	Generic
**Food Security**	Specific	Generic	Generic
**Nutrition**	Specific	Generic	Specific
**Shelter and settlement**	Specific	Generic	Generic
**Camp coordination and camp management**	Specific	n.a.	Generic
**Early recovery**	Specific	n.a.	Generic
**Education**	Specific	n.a.	Specific
**Protection**	Specific	n.a.	Specific

Source(s): (Global WASH Cluster 2016, 2017; IASC Reference Group on Gender and Humanitarian Action, 2018; *Minimum* *Standards* *on* *Gender:* *Food* *Security,* *Livelihoods* *and* *Cash* *Programming*, 2016; Shelter Cluster 2016; Sphere Project, 2018; The SEEP Network 2017; WHO, 2017a; WHO, 2017b; WHO, 2017c; WHO, 2020; World Vision, 2012)

For example, the IASC Gender Handbook health sector chapter contains a list of specific topics and questions for each response phase, including collection of SADD, the health situation of the affected population by sex before and since the crisis, household health care roles and responsibilities by sex, barriers to access, cultural expectations, and services for survivors (IASC Reference Group on Gender and Humanitarian Action 2018). However, it is over 400 pages long, which is overwhelming for users. As one key informant pleaded, “Just tell us the 5-10 things that must be done – there is no time to read all the guidance” (UN12).^5^ Similarly, the health cluster resources provided by the WHO, the cluster lead agency, call for the collection of disaggregated data, yet the overall guidance on programmatic interventions is too generic.

##### Inadequate gender expertise

Many in the humanitarian sector have determined that technical guidance alone is insufficient and that experts are also needed to support staff in addressing gender differences and inequalities. To meet this demand, OCHA established the Gender Standby Capacity Project (GenCap), an initiative that deploys gender advisors on short-term assignments to support humanitarian country teams. In the words of a UN professional, “One of the fundamental challenges about gender mainstreaming is that you do need someone with technical expertise, and it is more successful when we have those colleagues who are focal points or have that expertise within an operation, rather than it being everyone’s responsibility. When we have the earmarked funding to support gender specific expertise within an operation or region, it does make a difference” (UN5). Further, key informants conveyed that the uptake of gender equality approaches depends heavily on individual “champions” with the willingness to seek out and apply available resources (OE1, OE2, UN4). “In policy, [gender equality] was there always. In practice, it very much depended on personalities of who were driving the various initiatives. [Organizations] had their default positions, which on paper look quite strong, but I’d say it came down to personalities in terms of how it was carried forward” (OE2).

##### Insufficient financial resources

UN OCHA’s Financial Tracking Service (FTS) captures data on humanitarian funding flows. An analysis of that data has found that the amount of funding requested for activities with a focus on women and girls has increased over the years (UN Women and UNFPA 2020). However, the amount of funding allocated is far less than what is both needed and has been requested by humanitarian agencies (UN Women and UNFPA 2020; Chicey C, et al., 2022). Humanitarian assistance for gender equality and the empowerment of women typically falls under the protection and health sectors, or GBV as a separate stand-alone category. Occasionally, financing for activities to promote gender equality is also allocated to the agriculture, food security, and the water, sanitation, and hygiene clusters. The amount allocated is generally far less than the amount requested: 43% for reproductive health; 50% for child protection, and 33% for GBV (UN Women and UNFPA 2020). Moreover, programs that specifically target women and girls have the lowest levels of funding (Hatcher-Mbu 2021).

We conducted a deeper dive into the financing for GBV since protection of the most vulnerable is a priority for humanitarian response. Our analysis of the program documents shows that even when GBV was included within protection as a need in HNOs and addressed in the HRPs, it was not resourced robustly. As Table 5 shows, three of the eight country appeals (Bangladesh, Nigeria, Syria) requested funds to address GBV (FTS 2019a, b, 2020a). The funds they received ranged from 16% to less than half of the request. For the other five countries, there was no separate budget line item for GBV (FTS 2019c, d, 2020b, c, d). Moreover, despite high-level commitments to “localization” (UNGA 2016), financing for local organizations with expertise on GBV is reported to be scarce (IRC and VOICE 2019).

**Table 5:** Analysis of humanitarian funding for gender-based violence (GBV)

**Country**	**Appeal year**	**Total requested for GBV**	**Total funds received for GBV**	**% of funds received from amount requested for GBV**
**Afghanistan**	2019	n.a.	n.a.	n.a.
**Bangladesh**	2020	$24m	$3.9m	16.20%
**Colombia**	2020	n.a.	n.a.	n.a.
**Nigeria**	2019	$38.2m	$8m	20.80%
**South Sudan**	2019	n.a.	n.a.	n.a.
**Syria**	2019	$59.6m	$28.1m	47.20%
**Venezuela**	2020	n.a.	n.a.	n.a.
**Yemen**	2019	n.a.	n.a.	n.a.

Source(s): (FTS 2019d, c, b, a, 2020d, c, b, a)

### Humanitarian culture and behavior: a barrier to change

While mainstreaming has its own limitations, it is difficult to expect it to work in the context of humanitarian culture. Research in social psychology, evolutionary anthropology, and organizational science has shown that individual behavior and decision-making is non-conscious, often guided by mental short-cuts, and more influenced by organizational culture than any process-based bureaucratic interventions (Hallsworth and Kirkman 2020). Because culture is so important to individual behavior and decision-making, we analyzed our data through the lens of the behavioral science literature to understand the nature of humanitarian culture. We identified three dimensions of humanitarian culture that are particularly antithetical to promoting gender equality: a savior mentality and macho culture of a male-dominated field, a tolerance for abuse, and the short-term nature of crisis response and 12-month program cycles.

#### Savior mentality and a macho culture

With a focus on saving lives and distributing goods and services in dangerous and highly volatile circumstances, humanitarian actors typically display what has been described in the literature as “savior complexes” (Vijfeijken 2019). The passion to save lives and protect people from harm is deeply felt and imperative, but the protectionism and paternalistic attitudes that often drive this can be harmful to women. It perpetuates the focus on women’s and girls’ basic needs for food, shelter, and health and their vulnerability to gender-based violence, noted in the findings, with less attention to providing livelihood opportunities or appointing women to decision-making positions in humanitarian programs.

The need for change in the macho culture was a major theme raised by key informants (NGO6, NGO7, NGO9, UN4, UN9, UN13). As one key informant described it “[The rhetoric is] we must get in there and “save lives” because we’re the heroes and cowboys” (UN6). Key informants depicted humanitarian actors as driven by adrenaline and characterized by macho and masculine attitudes, making it difficult to have meaningful conversations about addressing gender differences in response (NGO5, NGO7, NGO8, UN6, UN13).

“[The organization was founded by] actors who were adrenaline junkies. They ran ambulance services, prehospital care, clinical services. Now that humanitarian action has blended so much with development [action], when I look at who is leading relief efforts and departments, they are largely men who come from search and rescue backgrounds. Conversations on [gender differences] are really hard to have” (NGO7).

An additional priority for humanitarian actors is to establish control in chaotic conflict settings. This establishment of control is a characteristic of the “macho” or “cowboy” culture that has been identified in the literature as a key feature of humanitarian action (Spencer 2018; Berdahl et al. 2018; Vijfeijken 2019). This is very similar to military culture, as explained by a key informant, because “when you’re dealing with conflict, your main interlocutors are military and paramilitary organizations and so many of our staff come from military backgrounds. You can see how the group thinking comes from a militarized, masculine viewpoint” (NGO5).

The cowboy macho culture, when combined with the inequality inherent in humanitarian situations between those who have the resources to distribute and those who are in need, contribute to an environment in which the abuse of power, including sexual exploitation and abuse, is more likely to happen, causing harm to both women and men (Spencer 2018; Berdahl et al. 2018). Although the humanitarian sector has begun to implement reforms to prevent sexual exploitation and abuse, some experts have expressed pessimism about such efforts taking root in organizations that place a high value on a “take charge,” “cowboy” style of leadership, where certain behaviors “which amount to bullying or harassment will be overlooked… [because] the person exhibiting that behavior otherwise is seen as a “hero” within the organization” (Vijfeijken 2019). As Vijfeijken (2019) notes, in a value-based sector like the humanitarian sector, “where passion for the cause is prized, aggressive work styles can be misinterpreted as signs of passion for the cause.”

Key informants identified several challenges to changing the cowboy culture, including the lack of female leadership and female staff in the humanitarian sector. A 2019 report reveals that only an estimated one third of Humanitarian Coordinators (HCs) are women and in 2020 that proportion improved only slightly to a little over one third (IASC 2019). This is not surprising since men were 65% of the pool of contenders for the HC position in 2020. In the words of one key informant “We have a real problem with the number of female staff to male staff in the higher echelons. We’ve set targets to reach but they’re hard to reach because the system keeps on pushing women back. It’s not only a matter of will but it’s a matter of systemic issues” (NGO5).

The preponderance of men on the staff of humanitarian organizations has consequences. Female informants explained that male colleagues do not fully understand the extent to which gender differences and inequalities matter. In the words of one interviewee: “The men never think about the fact that women are afraid or have to protect themselves because they’ve never had the same concern. To have that discussion when you go to a new location, what are the things you do to protect yourselves, their list would be empty. Then you have the female staff and their list would be filled with things to do to protect themselves, including not talking to strangers, and not going out alone at night” (NGO8). Another key informant stated:

“[My organization] is still 80-90% men. All the people I had to deal with were men. I was an international female staff member alone with a very male audience. There was an instance when someone just laughed at me when I said something about being a mother and how important it is to listen to mothers” (UN9).

Many argue that having more women humanitarian leaders will transform the sector, but for this to happen, male-normed leadership models cannot continue to be rewarded (Daly 2005; Ravindran and Kelkar-Khambete 2008; Madsen 2011; Davids et al. 2014; Mukhopadhyay 2014). As research has shown, without a shift in the traditional male-oriented model of leadership, women leaders are likely to experience an incongruity between cultural stereotypes of women and “effective” leadership (Eagly and Karau 2002). As a result, women are placed in a double bind—when they adhere to the expectations associated with their gender roles, they are likely to be viewed as ineffective leaders but when they are seen as competent leaders, they are criticized for not fulfilling their roles as women (Koenig et al. 2011).

#### A humanitarian culture that tolerates abuse

As noted above, in a culture that perpetuates controlling, paternalistic and protectionist attitudes, especially when combined with the inequality that is inherent between humanitarian actors and the communities they serve, the risk of abuse of power and exploitation of the weak is heightened. The sector has only recently begun to come to terms with the sexual exploitation and abuse (SEA) perpetuated by humanitarian workers against the people they serve.^6^

In addition to the disproportionate number of men as compared to women on the staff and in leadership positions of humanitarian organizations, which is one reason identified by the key informants for a culture that tolerates abuse (NGO4, NGO5, NGO7, NGO8, UN6, UN9), other factors cited by key informants were the lack of strong mechanisms of accountability, particularly in field operations in remote settings. Some informants argued that in settings in which humanitarian actors work round the clock, under pressure to save lives and with limited opportunities for rest or recuperation, the risk of exercising poor judgment is very high. They explained that although exposure to a challenging context does not justify unacceptable behavior, it underscores the need for robust supervisory and accountability measures (OE7, UN4, UN9, UN13).

#### Short-termism

A third aspect of humanitarian culture, as our findings revealed, can be called “short-termism.” The focus is on meeting the immediate needs of communities in crisis in the short-time available to respond (Vijfeijken 2019), as well as the short, discrete contract and funding cycles available for support. As one humanitarian professional noted: “It’s traditional that the humanitarian sector has different processes than the development sector – short-term, much shorter-term, sometimes a year or less for the appeal” (GDA4). As a result, another key informant explained “How do you do gender transformation in 12-month funding cycles? Well, you don’t if you only have funding for that period. So, the question is how can you get longer-term funding and resources to invest in staff, time, and partnerships to really make those changes?” (UN6).

Short-term funding cycles result in high staff turnover, including those who work to promote gender equality. This places a high burden on long-term staff to mobilize resources to replace the short-term expertise (IFI3, NGO7). One humanitarian worker elaborated, “It’s difficult because we’ll have the Protection, Gender and Inclusion Officer and the Gender Focal Point until project funding runs out. When their funding or secondment is up, we will once again search for a [member] that is willing to invest in gender [differences]” (NGO7). However, key informants themselves observed that new skills and strategies are needed to move away from rapid response operations to longer-term program cycles (GDA4, GDA6, NGO4, NGO9, OE7, UN4, UN5, UN6).

Because humanitarian actors are most concerned with producing immediate impact, investing in gender equality in contexts where it is not the norm can be perceived as hampering the ability to respond quickly (Hermann and Pagé 2016). In the words of one key informant, “It’s tricky if you actually want to implement social norms change programming because it requires a long-term perspective. It is challenging to plan longer-term projects that run over multiple years within the regular humanitarian program cycle.” (UN5)

This “short-termism” also makes it challenging for humanitarian actors to implement actions that bridge the humanitarian and development nexus—such as necessary economic and livelihood interventions—which lay the foundations for stronger and more resilient societies in which women and men have equality in capabilities, opportunity, safety, and agency.

## The way forward

Below we suggest a way forward, drawing from the conversations with gender experts and humanitarian professionals in the sample of this study and insights gained from behavioral science evidence and organizational change theory, as well as from our own professional experience in integrating a response to gender inequality in a range of development organizations. We do not claim to have all the answers but hope to trigger interest in a new way of working that could be as applicable to humanitarian organizations as they are to development agencies, despite the unique characteristics of each.

### Replace gender mainstreaming with a results-focused approach

First, we suggest replacing mainstreaming with a focus on results to be achieved for women and men, girls and boys, in specific sectors of humanitarian response. While the IASC Handbook for GEEWG lists expected results and good practices for achieving those results for each sector, they are placed in two different locations in the Handbook, making it difficult to match the action with the result. A simple first step would be to excerpt the expected results for each sector from the Handbook and match them with the action required, thereby increasing the specificity and accessibility of the guidance, with a clear link between actions and results. For example, for cash-based interventions, the expected result “women in need in targeted communities are able to access cash transfers without male assistance” which is on page 107 of the Handbook, should be paired with the good practice on page 114 “verify that women and men have equal access to mobile phones, bank accounts and identification cards.” While this recommendation is specific to the IASC, all organizations that produce or adapt guidance should identify concrete results and link them to the actions that can achieve them.

Second, we recommend against the use of jargon. Phrases such as “adopt a gender lens,” “incorporate gender considerations into,” or “implement gender transformative programs” are insider jargon and ambiguous. It is better to be more explicit and state in concrete terms the issue to be targeted, for example, provide women with a role in decision-making or reduce the gap between women and men in ownership of an asset, such as a parcel of land or a house by ensuring individual or joint title. In fact, to the extent possible, it is best to avoid the use of the term “gender” as a stand-alone term and instead specify whether the issue is to the disadvantage of women or men, girls or boys, or different segments of the population.

Third, gender analysis should be more diagnostic in nature and tailored to priorities in each program phase. As the IASC Gender Handbook recommends, at the initial stages of a crisis, a rapid assessment is appropriate and good for assessing the basic needs of women and men. Regrettably, however, it has become a “tick-the-box” exercise, too descriptive to be of use in program planning and implementation. To be useful, especially in the later stages of a crisis, humanitarian programs should move beyond a generic analysis to a more targeted diagnostic of the specific constraints and results to be achieved, especially the economic and agency constraints, that can be addressed through programs.

Fourth, complementing the already strong attention to girls and women’s basic needs and protection of affected populations with an equally robust investment in preventing and responding to GBV and increasing women’s economic and decision-making opportunities is essential, particularly as the humanitarian response shifts to recovery and resilience building. Financing for services and specialist staff trained to address GBV should be scaled up to be commensurate with the need and include support for local and national organizations. Equally important is improving the reporting and tracking of financing for GBV, as a separate category within the protection sector.

Further, use every opportunity to break out of gender-stereotypical programming, which sees women as mothers and caretakers and not economic actors, and men as providers but not caretakers. Efforts can be made to provide women with non-stereotypical economic opportunities such as job skills training programs for installation and maintenance of water pumps and solar energy or electrical repairs. Interventions can also lay the foundation for new gender roles that promote different norms of masculinity, for instance, involving fathers in parenting and household nutrition classes. Social safety nets and cash transfer programs that target women can be enhanced with productive economic inclusion^7^ components, including skills training or coaching, access to finance/savings, and links to market support (Andrews et al. 2021). Such programs not only allow households to invest in health, education, and nutrition, they also help prevent the renewal of conflict, as demonstrated through recent randomized trials in the Central African Republic, Cote d’Ivoire, Egypt, Liberia, and Uganda (World Bank Group 2015). Other evidence from cash transfer programs shows that alleviating income constraints can reduce the incidence of gender-based violence (Buller et al. 2018). Additionally, including girls and women in key decision-making roles, such as on the camp management committees or in monitoring processes that seek feedback from refugees, helps to build their agency and set new gender norms.

For some organizations, a results-based approach to promote gender equality may be seen as antithetical to the humanitarian principles of humanity, neutrality, impartiality, and independence, as codified in various UN General Assembly Resolutions (Bagshaw 2012). A strict adherence to these principles prevents organizations from making investments in longer-term development or disrupting gender norms since doing so is perceived to violate existing political and social structures.^8^ Moreover, as (Santos, 2020) argues, recent trends in humanitarianism prioritize human rights over local cultures that violate the rights of women and girls. This principle of upholding human rights over respecting cultures (by adhering to neutrality and impartiality) is featured in more recent organizational codes of conduct, either explicitly or implicitly, such as in UNHCR and Oxfam.

Ultimately, sustaining an effective response to closing gender gaps and empowering women by humanitarian organizations requires a change in the mind-set and behaviors of the individuals that make up those organizations. It is time to admit that the traditional approaches for ensuring compliance associated with gender mainstreaming, such as mandatory gender training, the use of gender markers, gender guidance and tools, and the appointment of gender experts to “police” gender integration—have not worked. Unswerving attention to the identification and prioritization of concrete and measurable results—without the clutter of processes and systems that obscure what changes or fails to change—is needed. It is time to let go of gender mainstreaming and experiment with new ways of doing business.

### Adopt behavioral strategies to support a results-based approach for gender equality

Highlighted below are some insights borrowed from the behavioral science and organizational change literature for gender experts to implement a results-based approach in humanitarian organizations. Some of these insights are already being tried by some of the key informants we interviewed. We list them here as suggestions of a way forward—a new approach to be tried and tested.

As stated before, much of an individual’s behavior is non-conscious, more often guided by “mental short-cuts” influenced by institutional norms, and what others are doing. For this reason, when faced with a choice, behavioral nudges toward a default option has a greater likelihood of changing behavior (Hallsworth and Kirkman 2020). Such automatic, habitual behavior is probably truer for humanitarian professionals who are required to make quick judgments and decisions when responding to a crisis, particularly in the early stages, with no time to consult detailed handbooks or conduct a separate gender analysis.

In light of this, our first suggestion is to nudge humanitarian actors into doing what is necessary by providing clear priority actions as the default minimum that must be addressed and that require an explanation for opting out of doing so.

For example, developing an app for every humanitarian worker’s mobile phone that lists the priority actions for each sector from the IASC Gender Handbook as the default option and matching them with the results to be achieved (as suggested above) could serve as the right nudge. In refugee camps, actions that could lead to transformative change may include providing identity documents to both women and men (the latter of whom are presumed to be the decision-makers), putting debit cards in the hands of women, and training women in water pump repair, electrical appliances, and vehicle maintenance. Setting these actions as the default, with the need to justify a decision to go “off script” and *not* implement them, may serve as the right behavioral strategy to implement effective interventions for gender equality.

Second, the organizational change literature suggests that a policy-led approach that offers guidance and capacity building to bring about change in humanitarian organizations is not the magic bullet it is thought to be, in part, because humanitarian professionals prefer more social learning approaches, such as hands-on, on-the-job training, and mentoring (Clarke and Ramalingam 2008). When the world is perceived in short, unconnected cycles, as humanitarians often do, it makes it harder to take the time for reflection, learning, and change. A “learning by doing” approach, such as through before- and after-action reviews, has the potential over traditional gender training methods to deepen impact by demonstrating the “what” and the “how” of addressing gender inequalities in real time, with practical examples.

Third, an analysis of humanitarian settings identifies two significant biases, cognitive and motivational, in decision-making (Comes 2016). It is thought that cognitive biases can be reduced by providing accurate information, expert advice or technical support, as gender mainstreaming has sought to do. However, Comes (2016) argues that such an approach is ineffective because cognitive biases are deeply rooted in motivational biases that maintain the status quo and existing power structures. Gender mainstreaming has failed because it does not tackle motivational biases.

Clarke and Ramalingam (2008) support this insight, suggesting that change is most effective when it builds on the intrinsic motivation of humanitarian professionals to reduce suffering and prevent harm. Demonstrating the value of gender equality and the empowerment of women in reducing suffering and preventing harm is more likely to be effective than the mere provision of technical information and guidance, especially because success begets success and may also help override motivational biases.

Humanitarian professionals, like development professionals, are also motivated by getting the funds they need to meet the needs of the communities they serve and as a result, a powerful way to incentivize humanitarian workers is by having donors link funding to achieving specific results to close gender gaps. It is important to not reduce those results to processes completed but to actual outcomes such as reductions in the incidence of child marriage or increases in women’s economic participation as compared as men.

Finally, peer effects are important because as individuals, we are strongly influenced by what our peers do (Hallsworth and Kirkman 2020). A first step is to identify influencers at all levels of the organization, who do not identify as gender experts, but have broad networks and can promote this agenda. Having those influencers provide hands-on support to their colleagues is more likely to be successful in seeding the approach across the organization than gender experts “policing” program behavior.

### Build a new humanitarian culture that promotes gender equality and the empowerment of women

Walking the talk on gender equality requires an enabling organizational culture and reforming organizational culture involves several elements. Bringing more women into humanitarian organizations and in leadership positions is an important first step to change the organizational culture. Proven ways to increase the recruitment of women include setting targets for women on staff, the board and management; ensuring diversity in the recruitment pool of candidates; and adopting measures to make sure evaluation criteria for selection are unbiased, such as by promoting blind reviews of applications and structured interview schedules to prevent unconscious bias (Lucas et al. 2021). Once women are hired, retaining them requires building an enabling environment, through supportive workplace policies such as parental leave, prevention and protection against sexual harassment, and family-friendly rotation and placement policies (Coury et al. 2020). Assuring the retention of female staff will help create a pipeline for promotion and advancement of women into leadership positions.

However, women leaders should not be expected to solve all the problems of the organizational culture—they are not superheroes. Having more women in leadership positions alone is not enough to change the humanitarian culture, especially if organizations reward a model of leadership that condones or actively promotes toxic masculinity (Berdahl et al. 2018; Vijfeijken 2019). The leadership model for both men and women needs to change to be more collaborative and transparent to cultivate the trust of staff, without compromising on achieving results (Koenig et al. 2011).

Recently, some multilateral organizations have begun working with third parties to verify whether these kinds of reforms are changing culture and creating a positive workplace environment for women and men. Third party verification, the results of which are independent and public, can be an important accountability mechanism.^9^ Humanitarian organizations committed to change might consider using this approach.

Individual organizations, however, cannot do it alone. The change in culture needs to be adopted by the entire humanitarian system and reinforced by donors. It requires work of “a long-term, sustained and disciplined nature,” focusing on the “encouragement of habitual and widely shared behaviors that nurture self-awareness and humility” together with the courage to call out people who do not follow the new norms (Vijfeijken 2019).

## Conclusion

Our analysis finds that gender mainstreaming approaches in humanitarian organizations fall short of achieving the IASC goals of gender equality and women’s empowerment. We uncover factors driving this failure, including aspects of humanitarian culture. The community of “gender experts” of which we are members, is part of the problem because of our insistence on using an approach that has not led to the transformational change that we aspire to. In this paper, we have suggested the ingredients of an alternative approach to increase the effectiveness of humanitarian response for enhancing capabilities, opportunities, safety, and agency of girls and women, as well as ways to transform organizations. Nowhere is an alternative approach more important than in the humanitarian sector.

## Data Availability

Data for this study was drawn from publicly available organizational documents listed in Annexes 1–2 and key informant interviews with 44 individuals from 15 global organizations. All data analyzed during this study are included in this article and its annexes.

## Appendix 1

**Table:** **Table 6** Analytical Framework for Analyzing Key Informant Interviews

Theme	Code	Description
Organizational Elements and Processes	Leadership commitment	References to commitment from senior leadership and management or clear motivations for adoption by senior leadership
	Policy/strategy	Organization document(s) that outline commitment to gender equality and strategy or approaches for the organization
	Staffing structure and reporting lines	Gender focal point, gender advisors, staffing allocations
	Funds	Specific fund for gender programming, earmarked funds
	Training and guidance/tools	Training modules, frameworks, toolkits, guidelines, other guidance for how to implement the approach
	Evidence on strategic entry points and ways to intervene	
	Incentives	Motivations outlined at the organization to encourage staff to integrate gender
	Monitoring mechanisms with indicators	Monitoring and evaluation frameworks, indicators that measure gender specific outcomes
	Accountability mechanisms	Project reviews, Programming checklist, monitoring and evaluation frameworks,
Key Ingredients for Success	Political commitment and buy-in	Gender equality recognized on the national agenda of partner countries and/or by partner organizations
	Technical experts and resources	Gender advisors, gender focal points, experts with cross-sectoral expertise, training modules
	Time	Length of time organization has been committed to gender equality, staffing allocations, budget allocations, review meetings, conferences, partnership collaboration
	Funds	Earmarked organization funds, partnership funds
Strategy/Approach Continuum	Process-oriented vs. Outcome-oriented	Focused more on the policies and strategies leading up to an approach or on the actual outcomes of implementing an approach or both
	Parity as priority vs. Program as priority	Focused on increasing gender equality within organizational staff or within programming planning and implementation or both
	Practical vs. Strategic	Focused on the practicality of achieving outcomes now or a long-term planning strategy
	Instrumentalist vs. Rights-based	Needs-based or understand everyone has basic human rights and approach by focusing on those most excluded/marginalized by analyzing gender norms and other forms of discrimination
	Cookie-cutter vs. Context specific	Use the same approach in every program/context or revise and adapt approach to consider context-specific factors

## Appendix 2

**Table:** **Table 7** Gender Policy, Analysis and Technical Resources by Institution

Organization	Policy	Is a gender analysis recommended?	Sample Technical Resources
Office of the Coordinator for Humanitarian Affairs (OCHA)	-OCHA Policy Instruction on Gender Equality 2016-2020 -Inter-Agency Standing Committee (IASC) Policy on Gender Equality and the Empowerment of Women and Girls (GEEWG) in Humanitarian Action	Yes	- IASC GEEWG Accountability Framework - IASC Gender Handbook for Humanitarian Action - IASC Gender with Age Marker - eLearning course on Gender Equality in Humanitarian Action - IASC Guidelines for Integrating Gender-based Violence Interventions in Humanitarian Action
The UN Refugee Agency (UNHCR)	UNHCR Policy on Age, Gender and Diversity	Yes	- Instructions for Field Officers concerning refugee women and children - People Oriented Planning Training Strategy - Gender Training Kit on Refugee Protection and Resource Handbook - UNHCR Gender Equality Toolkit - Preparedness Package for Refugee Emergencies
United Nations Children’s Fund (UNICEF)	-Gender Action Plan 2018-2021 -Core Commitments for Children in Humanitarian Action	Yes	- Core Commitments for Children in Humanitarian Action
World Food Program (WFP)	WFP Gender Policy 2015-2020	Yes	- WFP Gender Toolkit - WFP Gender and Age Marker - WFP’s Gender Transformation Programme: Office Guide
World Health Organization (WHO)	-Strategy for Integrating Gender Analysis and Actions Into the Work of WHO - Thirteenth General Programme of Work 2019-2021 (GPW13)	Yes	- WHO Gender Mainstreaming Manual for Health Managers: A Practical Approach - Innov8 approach for reviewing national health programs to leave no one behind Integrating Equity, Gender, Human Rights and Social Determinants Into the work of WHO: Roadmap for Action 2014-2019 - Strengthening Preparedness for Health Emergencies; Implementation of International Health Regulations - Emergency Response Framework - Standards for Public Health Information Services - Operational Guidance on Accountability to Affected Populations
Global Affairs Canada	Policy on Gender Equality Canada’s Feminist International Assistance Policy	Yes	- Mainstreaming of a Gender Perspective - Framework for Assessing Gender Equality Results - Feminist International Assistance Gender Equality Toolkit for Projects - How Projects are Coded for Gender Equality - Canada’s National Action Plan 2017-2022 - Integrating Gender Equality Into Programming - A Feminist Approach: gender equality in humanitarian action
Swedish International Development Cooperation Agency	The Swedish Foreign Service action plan for feminist foreign policy 2019-2022	Yes	- Gender Mainstreaming Tool Box - Strategy for Sweden’s humanitarian aid provided through the Swedish International Development Cooperation Agency (Sida) 2017-2020 - Gender Equality in Humanitarian Assistance
USAID	Gender Equality and Female Empowerment Policy	Yes	- Measuring Gender Integration in USAID Planning Procurement - Integrating Gender Equality and Female Empowerment in USAID’s Program Cycle - Women’s Entrepreneurship and Economic Empowerment Act of 2018 - USAID’s Women, Peace, and Security Implementation Plan
African Development Bank	Gender Policy 2001	No^a^	- Gender Mainstreaming at the African Development Bank Group: A Plan of Action - African Development Bank Group Strategy for Addressing Fragility and Building Resilience in Africa 2014-2019
European Bank for Reconstruction and Development	Strategic Gender Initiative	Yes	- Strategy for the Promotion of Gender Equality 2016-2020 - The EBRD refugee response plan
Inter-American Development Bank	Operational Policy on Gender Equality in Development (2010)	Yes	- The Gender Action Plan (2017-2019)
International Federation of Red Cross and Red Crescent Societies	IFRC Gender Policy (1999)	Yes	- IFRC Strategic Framework on gender and Diversity Issues 2013-2020 - Gender and Diversity Organisational Assessment Toolkit - Minimum standards for protection, gender and inclusion in emergencies - Training on Protection, Gender and Inclusion in the International Federation of Red Cross and Red Crescent Societies - Protection, Gender and Inclusion in Emergencies Toolkit: Pilot Version
International Committee of the Red Cross	None identified	No	None identified
International Rescue Committee (IRC)		Yes	- IRC Gender Equality Scorecard and Action Plan - Outcomes and Evidence Framework - Good and Great Standards - The Essentials for Responding to Violence Against Women and Girls During and After COVID
CARE	CARE International Gender Policy	Yes	- Gender Equality and Women’s Voice Guidance Note - CARE International Humanitarian and Emergency Strategy 2013-2020 - When Time Won’t Wait: An Evaluation of CARE’s Rapid Gender Analysis - Gender is Easy: A guideline for doing a gender analysis in emergencies - CARE International’s Strategic Impact Inquiry on Gender in Emergencies: Pilot Summary Report - Strategic Impact Inquiry on Gender in Emergencies Phase Two: Initial Steps & Ways to contribute

^a^AFDB’s fragility analysis tool includes a section on gender, but there is no evidence of recommending a gender analysis from key informants or organizational documents.

## Footnotes

1 To ensure confidentiality, key informants were assigned a serial number within each category of type of organization as shown in Annex 1. We use the assigned serial number to cite the key informants in the text of the paper.

2 UN Women and UNFPA were not included in the sample because their core mission is to reduce gender inequality and empower women and girls and hence, they serve as the expert agencies within the UN on gender equality.

3 Admittedly, collecting sex and age disaggregated data in humanitarian contexts is challenging and data are already limited because the governments have not had the capacity or tools to collect it.

4 Clusters are groups of humanitarian organizations, both UN and non-UN, in each of the main sectors of humanitarian action, e.g., water, health and logistics. They are designated by the Inter-Agency Standing Committee (IASC) and have clear responsibilities for coordination.

5 Several key informants pointed out that while this is a common complaint, even when lists of priority actions are provided, they are not necessarily implemented.

6 SEA is defined as any actual or attempted abuse of a position of vulnerability, differential power or trust for sexual purposes or the actual or threatened intrusion of a sexual nature by UN personnel, their implementing partners or other aid workers (United Nations Secretary-General 2003).

7 Productive economic inclusion programs have been adopted in 75 countries and represent a potentially transformational approach in fragile and conflict settings (Andrews et al. 2021).

8 One example to illustrate this point is ICRC. Given its mandate to respond to needs arising from armed conflicts, and as a custodian of the Geneva Convention, ICRC has opted not to focus on gender equality or women's empowerment because such programming is perceived to be in violation of the principles of neutrality and impartiality.

9 Examples of such third party accountability mechanisms are Global Health 50/50 and EDGE Certification (EDGE).
